# The role of concern about falling on stepping performance during complex activities

**DOI:** 10.1186/s12877-019-1356-z

**Published:** 2019-11-27

**Authors:** Shaira Viaje, Geert Crombez, Stephen R. Lord, Jacqueline C. T. Close, Perminder Sachdev, Henry Brodaty, Kim Delbaere

**Affiliations:** 1 Falls, Balance and Injury Research Centre, Neuroscience Research Australia, University of New South Wales, PO Box 1165, Sydney, Randwick, NSW 2031 Australia; 2 Department of Experimental-Clinical and Health Psychology, Ghent University, Gent, Belgium; 3 School of Public Health and Community Medicine, University of New South Wales, Sydney, Australia; 4 Prince of Wales Clinical School, Medicine, University of New South Wales, Sydney, Australia; 5 Neuropsychiatric Institute, Prince of Wales Hospital, Randwick, New South Wales, Sydney, Australia; 6 Centre for Healthy Brain Ageing (CHeBA), School of Psychiatry, University of New South, Sydney, Wales Australia; 7 Dementia Centre for Research CollaborationUniversity of New South Wales, Sydney, Australia

## Abstract

**Background:**

There is limited understanding of the underlying mechanisms explaining the role of concern about falling on fall risk in older people. Anxiety is known to interact with cognitive resources and, as people get older, they require more cognitive resources to maintain balance. This might affect an individual’s ability to perform cognitive-motor tasks concurrently. The aim of this study was to investigate the effect of a visuospatial dual-task on stepping performance in older people with and without concern about falling and the impact of repeating this task in those with high concern about falling.

**Methods:**

Three-hundred-eight community-dwelling older people, aged 70 to 90 years old, participated in the study. Participants were asked to perform a Choice Stepping Reaction Time (CSRT) task in two conditions; once without any other tasks (single task condition), and once while simultaneously performing a visuospatial task (dual-task condition). Participants were asked to rate their levels of concern and confidence specifically related to each of the 25 stepping trials (before/after). We also measured general concern about falling, affect, and sensorimotor and cognitive functioning.

**Results:**

Total stepping reaction times increased when participants also performed the visuospatial task. The relation between general concern about falling and stepping reaction time, was affected by sensorimotor and executive functioning. Generalised linear mixed models showed that the group with moderate to high levels of general concern about falling had slower total stepping reaction times than those with lower levels of concern about falling, especially during the dual-task condition. Individuals with greater general concern about falling showed reduced confidence levels about whether they could do the stepping tasks under both conditions. Repeatedly performing the stepping task reduced the immediate task-specific concern about falling levels and increased confidence in all participants.

**Conclusions:**

These findings reveal that people with higher general concern about falling experienced more difficulties during a dual-task condition than people with lower levels of concern. Of further interest, better sensorimotor and cognitive functioning reduced this effect. Graded exposure has potential to reduce concern about falling during fear-evoking activities, especially in conjunction with therapies that improve balance, mood and cognitive function.

## Background

Concern about falling is common in older people, with prevalence rates up to 43% [1]. Falls can result in injuries which impose limitations upon daily activities, jeopardize autonomy and decrease quality of life [2, 3]. These devastating consequences of falls are possible reasons for the high prevalence of concern about falling in older people who have suffered a fall in the past. However, concern about falling is also reported in individuals without a fall history [4, 5]. A wide range of factors has been associated with concern about falling, many of which are also known risk factors for falls, such as being female, being older and having poor balance and gait [5, 6]. Additionally, impaired cognitive ability [7], depression and anxiety [8] have been associated with concern about falling. History of falls has been found to be a predictor for developing concern about falling [4]. For individuals who have never experienced a fall, concern about falling could develop through forecasting the possible consequences of falls [9].

Concern about falling may have paradoxical effects. High levels of concern about falling are associated with avoidance of daily activities [10]. Such avoidance behaviours can result in physical inactivity, decreased muscle strength and balance, which increase fall risk through deconditioning [11]. Concern about falling can increase fall risk, even in older people who do not have impaired balance or other obvious risk factors [12]. Previous studies have found that individuals with concern about falling often adopt a slow gait speed as a strategy to protect themselves from falling and maintain balance during high-risk activities [13, 14]. People who display such gait behaviour may, however, fall more because a slower gait reduces the individual’s stability and therefore increases the likelihood of falling [14]. Furthermore, concern about falling may also affect a person’s fall risk due to the interplay between anxiety and attention [15]. Anxiety may interfere with tasks that require attention and complex coordination. Therefore, concern about falling may further increase the challenge of tasks such as walking, especially under dual-tasking conditions [16]. As age increases, walking and maintaining posture becomes more cognitively demanding and less automatic [17]. Anxiety might then interfere with this process and increase the challenge to remain upright while walking [18–21]. There is limited understanding about how to reduce concern about falling. Cognitive behavioural therapy is a psychotherapeutic approach known to be efficacious in the treatment of depression and low mood, with lasting effects that protect against relapse following the end of the treatment [22] and may also be relevant to reduce concern about falling [10]. The aim of cognitive behavioural therapy is to target (erroneous) thoughts and beliefs. Exposure to fear-evoking stimuli has been used as a strategy in cognitive behavioural therapy and may be of particular use in our context. Wetherell et al. (2016) suggested graded exposure to fear-evoking tasks as a treatment for concern about falling. However, this research topic is still in its infancy and not well-understood.

The aims of this study were twofold. First, we explored the effect of cognitive task on the performance of a stepping task in individuals with and without concern about falling. Second, we examined how repeating this stepping task affected task-specific concern about falling - especially in those with higher general levels of concern about falling. We also attempted to investigate whether this relationship was affected by mood or executive function. Our experiment was informed by two theories. Attentional control theory posits that task performance will be worse when individuals cannot efficiently control their internal thoughts and feelings of anxiety [18]. According to the selective exposure theory, individuals tend to favour information that reinforces their anxiety, or information in their environment that is congruent with and confirms their current attitudes [23]. To the best of our knowledge, the current study is the first to apply these theories on dual-task performance in relation to concern about falling. More specifically, this study investigated the performance of older people in a stepping reaction time task, once in a single task condition and once in a dual task condition. We hypothesised that stepping performance during the dual-task condition will be worse in individuals with concern about falling compared to those with lower concern. Additionally, we hypothesised that task-specific concern about falling will be higher during the dual-task condition and will reduce after repeating the stepping task. Finally, due to the interaction between anxiety, depressive symptoms and executive resources, we hypothesised that the relationship between concern about falling and stepping performance will be affected by current mood or executive function.

## Methods

### Participants

Three hundred eight older adults aged over 70 years old were recruited from a sample of individuals in the eastern Sydney community participating in the first stage of the Sydney Memory and Ageing Study [24]. Participants were excluded if they had (i) a neurological disorder (i.e. dementia, Parkinson’s disease, multiple sclerosis, motor neuron disease or central nervous system inflammation), (ii) psychological disorder (i.e. psychotic symptoms, lifetime history of severe psychological conditions), (iii) developmental disability, (iv) self-reported inability to walk 20 m without a walking aid or (v) any other medical condition that may prevent them from completing the assessments that were required in the study. The protocol was approved by the Human Studies Ethics Committee HREC 05224 at the University of New South Wales and informed written consent was obtained from all participants.

### Experimental procedure

**Fig. 1:**
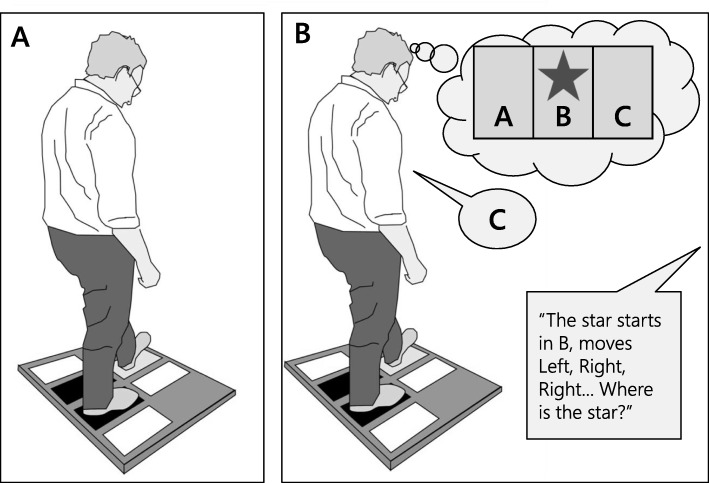
Experimental procedure: Panel **a** shows the Choice Stepping Reaction Time task as a single-task condition, Panel **b** shows the Choice Stepping Reaction Time task as a dual-task condition while simultaneously performing a visuospatial task

We used the same methodology as previously described by St George et al. [25]. Participants completed a Choice Stepping Reaction Time (CSRT) task under two conditions (with and without a visuospatial task). The CSRT device consisted of a low platform (84 cm × 76 cm) and six rectangular panels (32 cm × 12 cm): two base plates and four stepping plates. The participant stood on the base plates and stepped onto an illuminated stepping plate presented in a random order (Fig. 1). A supra-threshold was applied so that no participants had difficulty detecting the Target stimuli. Participants were instructed to step onto a plate as quickly as possible when it was illuminated, using the left foot only for the plates on their left (front and side) and the right foot only for the two right plates. Participants stood with their feet 12 cm apart and parallel with the two side plates. The light of the illuminated plate turned off when participants had stepped with their full foot. They then moved their foot back to the starting base plate at their own pace. Pressure switches under each plate recorded the time of stepping events to within 1-millisecond accuracy throughout a trial. There was usually between 5 and 10 s delay before the start of a new trial to allow participants to regain their balance. Response time was recorded as the time from the plate illumination to the foot lift-off of the appropriate leg. Transfer time was recorded as the time from the appropriate foot lift-off to foot contact on the correct plate. From the lift-off events recorded by the stepping plates, incorrect start data were obtained. Thus, this method recorded “correct” response and transfer times with any errors made in selecting the appropriate stepping foot included in the response time and any errors made in stepping to the target included in the transfer time. For example, if participants initially raised the incorrect stepping foot and then replaced it before initiating movement with the correct leg, these events increased the response time measure. If participants stepped with the correct leg but did not land on the correct plate and required an additional step to reach the target, this increased the transfer time measure. The total CSRT reaction time in milliseconds was used for all analyses: this comprised the sum of response and transfer times. All trials were included in the analyses, regardless of whether the participant made errors.

The CSRT task was performed either alone (single task condition) and while simultaneously performing a visuospatial task (dual-task condition). The visuospatial task was used because it allowed participants to look directly at the stepping plates of the platform while performing the primary stepping task and provide a record of cognitive task responses– allowing ascertainment of whether or not participants were attending to the secondary task. The visuospatial “star movement” task, adapted from Brooks [26], required participants to envision three boxes side by side labelled A, B, and C (Fig. 1). Participants were then asked to visualise a star in one of boxes and making three movements. They were told the starting box of the star and the direction of the three movements, i.e., left or right. Participants were shown a visual display during the explanation of the protocol. They were allowed sufficient practice without the visual display until they demonstrated that they understood the test requirements and could score 5 consecutive correct responses. For each trial in the dual-task, the experimenter verbally delivered the initial position and three movements of the star and immediately following this, a stepping plate was randomly illuminated. Participants completed the step and then reported the finishing box of the star, i.e., box A, B, or C. Performance was recorded in terms of the number of errors made in identifying the finishing position. We used the same methodology as previously described by St George et al. [25].

The two conditions were administered in a random order with each comprising 25 trials with five illuminations per plate. Each of the 25 CSRT trials for each condition, took 3 to 5 min to complete. To measure the effect of repeating this stepping task 25 times, participants were asked to indicate their levels of confidence to perform the stepping task and task-specific concern about falling 4 times, before and immediately after each condition on a 10-point Likert scale. Participants gave a rating between 1 and 10 (‘1’ represented lowest confidence, or greatest concern: ‘10’ represented highest confidence and least concern). Confidence questions were ‘how confident are/were you that you can/cou the stepping task without making errors?’ The question about task-specific concern about falling read, ‘to what extent are/were you concerned that you will/might lose your balance during the stepping task?’

### Other measures of interest

The Physiological Profile Assessment (PPA) [27] was assessed as a measure of sensorimotor function. The PPA is a validated composite measure of fall risk, containing five assessments: visual contrast sensitivity (Melbourne Edge Test), proprioception (measured with a lower limb-matching task), reaction time (measured using a light as stimulus and a finger-press as response), postural sway (path length measured with a sway meter recording displacements of the body at the level of the pelvis while standing on a foam mat with eyes open), and muscle strength (measured isometrically in the dominant leg with participants seated with the hip and knee flexed 90°) [27].

Falls Efficacy Scale International (FES-I) [28] was used to assess general concern about falling. Participants rated their concern about falling during 16 daily activities on a 4-point scale (‘1’ = not at all concerned; ‘4’ = high concern). The sum score may range between 16 and 64. A higher score reflects greater concern about falling. For this study, individuals were categorized using a validated cut-point, based on their levels of general concern about falling: group of low concern (FES-I scores ≤19) and group of moderate to high concern (FES-I scores > 19) [12].

Depressed mood and anxiety were assessed with two questionnaires. The Geriatric Depression Scale (GDS) is a 15 item instrument to screen for depression in older people [29]. Scores ranged from 0 to 15 with higher scores representing more depressive symptoms. Anxiety was assessed using the Goldberg Anxiety Scale (GAS) [30]. A high score on this 9-item questionnaire reflected greater anxiety.

Cognitive processing performance was tested with a trail making test (TMT) [31], more specifically as a measure of visual search and executive function including psychomotor speed, set-shifting and working memory. In part A, participants connected letters in ascending order. In part B, they were required to connect alternating letters and numbers in ascending order as quickly as possible. Set-shifting was measured as the difference in completing parts A and B. Maximum completion for part A was set at 180 s and 320 s for part B [32]. The TMT ‘executive function’ score was computed by subtracting Part A from Part B.

### Statistical analyses

The statistical software, SPSS Version 24 was used for all analyses and a value of *p* < 0.05 was considered significant. Linear regression was used to explore the strength of the association between CSRT performance and FES-I (univariable) and after controlling for PPA, GDS, GAS and TMT (multivariable). TMT measures of cognitive function were controlled for years of education in all analyses. Generalised linear mixed models were used to analyse differences in CSRT performances [1] between single and dual-task conditions, and [2] between people with low and high general concern about falling (FES-I scores ≤19 vs > 19). In addition, interaction effects between condition and groups were also examined. Similar models were used to analyse differences in task-specific confidence and concern about falling over time. Measures of PPA, previous falls, GDS, GAS and TMT were added separately as covariates to the analysis.

## Results

The mean age of our participants was 78.0 years (SD = 4.5) and 53% (*N* = 162) were female. Out of a possible seven common medical conditions, the sample had a mean of 3.1 (SD = 1.5) and 85% (*N* = 262) rated their health as good, very good, or excellent on a self-rated general health question using a five-point scale. With respect to falls information, 31% (*N* = 96) of the participants reported one or more falls in the previous year, and 60% (*N* = 185) reported moderate to high levels of general concern about falling (Table 1).

**Table 1:** Summary of descriptive data for total sample, participants with lower (FES-I ≤ 19) and higher (FES-I > 19) levels of concern

	Total sample (*N* = 308)		Lower Concern (*N* = 123)		Higher Concern (*N* = 185)	
	Mean	SD	Mean	SD	Mean	SD
CSRT Single Task (ms)	1007.0	205.7	974.9	203.8	1028.4	204.6
CSRT Visuospatial Task (ms)	1845.1	863.6	1668.2	743.3	1962.8	918.2
Concern about falling (FES-I *range 16–64*)	22.5	6.3	17.9	0.98	25.6	6.5
Mood (GDS *range 0–15*)	2.21	1.92	1.61	1.6	2.61	2.0
Anxiety (GAS *range 0–9*)	0.99	1.68	0.67	1.34	1.20	1.8
Processing speed (TMT A, s)	46.1	20.2	45.5	22.9	46.4	18.4
Task-switching (TMT B, s)	127.9	69.9	126.3	75.2	128.9	66.4
Executive function (TMT B – A, s))	81.9	61.7	80.9	66.4	82.5	58.6
PPA	0.907	0.910	0.711	0.816	1.03	0.948
MMSE	28.51	1.39	28.59	1.22	28.46	1.50
Previous falls in the past year	0.52	0.88	0.28	0.68	0.68	0.96

*Legend: CSRT = Choice Stepping Reaction Time, FES-I = Falls Efficacy Scale International, GDS = Geriatric Depression Scale, GAS = Geriatric Anxiety Scale, TMT = Trail Making Test; lower scores represent better performance in all tests*

Table 2 shows that FES-I, was significantly associated with average reaction times of the stepping task during both the single task condition and the dual-task condition. In the single task condition, the strength of the association was reduced by 37%, after controlling for PPA; yet both variables stayed significant, suggesting that both have a unique contribution in predicting the performance during the single task condition. In the dual-task condition, the strength of the association was also reduced by 38%, after controlling for PPA. However, only PPA remained significant. When TMT was entered in the model (instead of the PPA), the strength of the association of the FES-I was reduced by 19%; with both variables staying significant, suggesting that both variables have an independent contribution in predicting the performance during the dual-task condition. When GDS and GAS were entered in the model, the strength of the association between FES-I and CSRT was not significantly affected.

**Table 2:** Results of regression analysis, identifying the strength of associations between FES-I and stepping performance while controlling for PPA, GDS, GAS and TMT

	Single task CSRT		Dual-task CSRT	
	FES-I*#*	Covariate†	FES-I*#*	Covariate†
Concern about falling (FES-I)	6.59 (1.82), *p* < 0.001	–	18.04 (7.73), *p* = 0.020	–
Fall risk (PPA)	4.18 (1.74), *p* = 0.017	79.61 (12.13), *p* < 0.001	11.48 (7.72), *p* = 0.138	215.96 (53.68), *p* < 0.001
Mood (GDS)	5.78 (1.90), *p* = 0.003	10.54 (6.30), *p* = 0.095	15.89 (7.74), *p* = 0.041	43.46 (25.63), *p* = 0.091
Anxiety (GAS)	6.11 (1.89), *p* = 0.001)	5.06 (7.07), *p* = 0.475	18.06 (7.95), *p* = 0.024	11.56, (29.66), *p* = 0.697
Executive function (TMT)	6.05 (1.80), *p* = 0.001	0.72 (0.18), *p* = 0.313	14.69 (7.44), *p* = 0.049	3.52 (0.76), *p* < 0.001

*Legend: FES-I=Falls Efficacy Scale, GDS = Geriatric Depression Scale, GAS = Goldberg Anxiety Scale, TMT = Trail Making Task, B = regression weight, SE = Standard Error*

*# Univariable regression analysis between measures (FES-I, PPA, GDS, GAS, TMT) and stepping performance;* **†** *Multivariable linear regression analysis between measures FES-I and stepping performance, controlling for PPA, GDS, GAS or TMT*

**Fig. 2:**
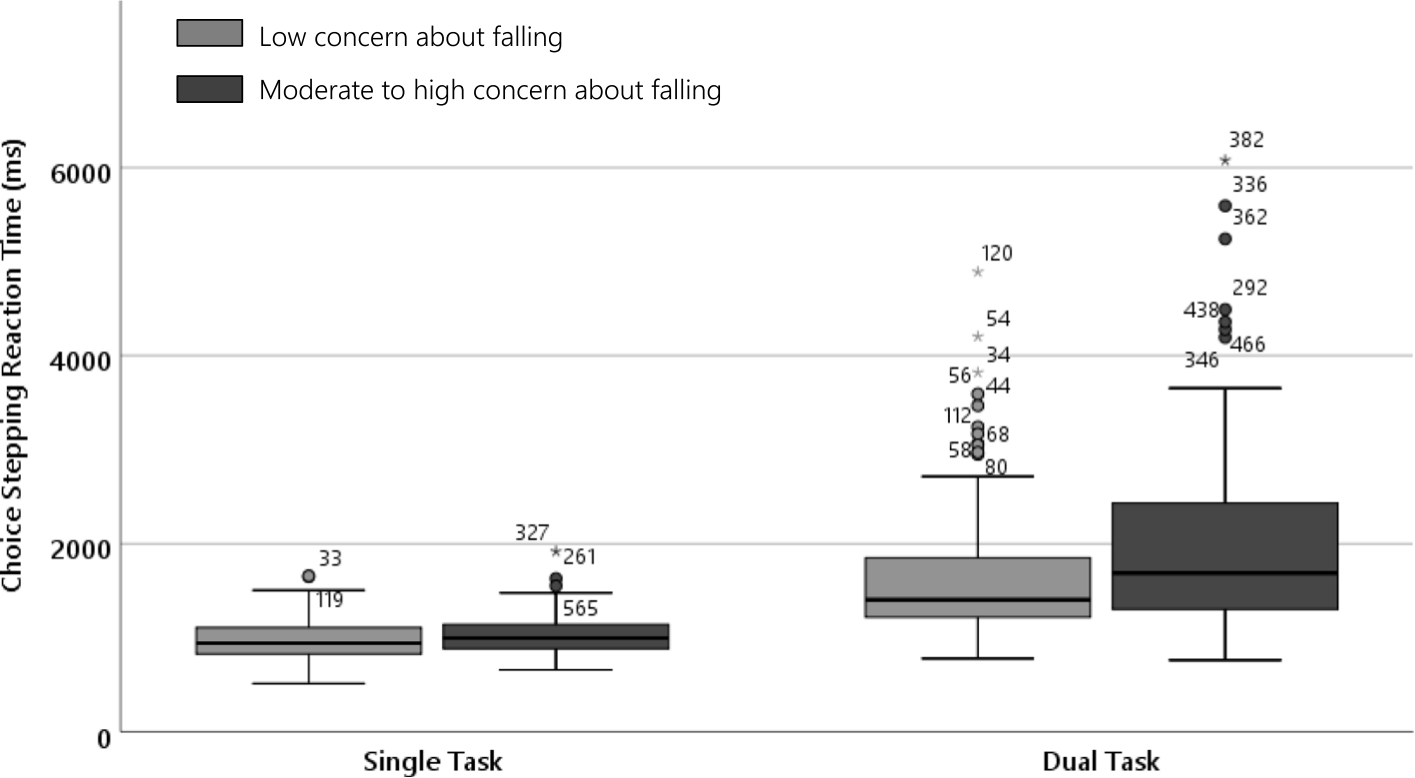
Boxplot of average total stepping reaction times for simple and visuospatial CSRT in participants with lower concern about falling and moderate to high concern about falling

Generalised linear mixed models confirmed that the total stepping reaction times were significantly longer during the dual-task condition compared to the single-task condition (β = − 934.4, 95% CI [− 1058.7 to − 810.1], *p* < 0.001) – see Fig. 2. The group with moderate to high levels of general concern about falling had slower total stepping reaction times than those with lower levels of concern about falling (β = − 294.6, 95% CI [− 480.8 to − 108.4], *p* = 0.002). The group × condition interaction was significant and indicated that the dual task affected the CSRT task performance more in the group with higher levels of general concern about falling (β = 241.2, 95% CI [70.8 to 411.6], *p* = 0.006).

**Fig. 3:**
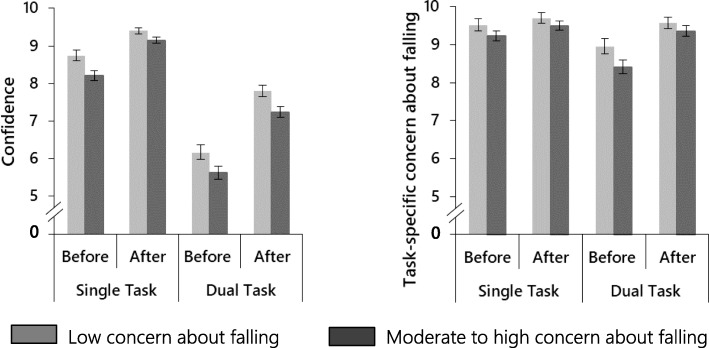
Responses to questions about feelings of confidence and concern about falling before and after each CSRT condition. Groups are based on FES-I scores. Low concern (FES-I ≤ 19) and high concern (FES-I > 19). Ratings made on a 10-point Likert scale with 1 – lowest confidence, greatest fear and 10 – greatest confidence, least fear. Error bars are presented as Standard Errors of the Mean

The effect of repeating the task on task-specific confidence and concern about falling is illustrated in Fig. 3***.*** Analysis of ratings before and after each condition revealed a significant difference in ratings between time (before vs after task) and condition. Participants became more confident (β = − 1.618, 95% CI [− 1.918 to − 1.317], *p* < 0.001) and less concerned (β = − 0.951, 95% CI [− 1.250 to − 0.652], *p* < 0.001) after the performance of the stepping task in comparison with before. Overall, participants were also more confident (β = 1.902, 95% CI [1.621 to 2.184], *p* < 0.001) and expressed lower levels of task-specific concern (β = 0.141, 95% CI [− 0.015 to 0.296], *p* = 0.076, borderline significance) in the single task condition than in the dual-task condition. Group differences were only found for confidence: the group with higher levels of general concern about falling were less confident regarding both conditions compared to the group with lower levels of concern (β = 0.554, 95% CI [0.135 to 0.972], *p* = 0.010). The significant time × condition interaction effects for ratings of confidence (β = 0.682, 95% CI [0.338 to 1.025], *p* < 0.001) and task-specific concern about falling (β = 0.676, 95% CI [0.318 to 1.033], *p* < 0.001) indicated adjustments towards higher confidence and lower concern about falling were more pronounced after repeated in the dual-task condition in comparison with the single task condition. The group × time interactions were not significant for either confidence (β = − 0.031, 95% CI [− 0.535 to 0.474], *p* = 0.905) or concern ratings (β = 0.325, 95% CI [− 0.077 to 0.728], *p* = 0.113). In a further series of exploratory analyses, we explored the role of generic measures of sensorimotor functioning, falls in the past year, cognitive functioning and depressed mood and anxiety on the group × time interaction. Controlling for PPA or previous falls had no impact on this interaction. However, the interaction became significant for task-specific concern about falling (β = 0.392, 95%CI [0.002 to 0.781], *p* = 0.049) when controlling for GDS; and borderline significant (β = 0.387, 95% CI [− 0.005, 0.778], *p* = 0.053; β = 0.386, 95% CI [− 0.002, 0.774], *p* = 0.051) when controlling for GAS and TMT. Further, post-hoc probing analyses [33] suggested that people with high general concern and low mood (GDS > 5) were less likely to adjust their task-specific concern ratings over time. However, caution is required when interpreting these results due to reduced sample sizes.

## Discussion

This study investigated the experience and performance during a stepping task under single task and dual-task conditions in older people with low and higher levels of general concerns about falling. The study also explored whether repeating the stepping task improved ratings of task-specific concern and confidence. The results confirmed that participants, overall, were slower during the dual-task condition, but, and of particular interest, the group with higher levels of general concern about falling were more affected in the dual-task condition than the group with low levels of concern. Results indicated that participants overall benefitted from exposure to the stepping task: the task-specific concern about falling diminished over trials. Further exploratory analyses revealed that poorer sensorimotor function and executive function affected the relationship between general concern about falling and the performance of stepping task in the dual task condition. These findings highlight the negative relationship between general concern about falling and stepping ability, especially during complex activities. Improved sensorimotor function and better cognitive function may reduce this impact.

### Processes explaining the relation between general concern about falling and performing complex activities

Previous studies have shown that performing dual-task activities becomes more challenging as people get older and a reduced ability to simultaneously perform multiple tasks might subsequently impact risk of falling [14, 34]. Our results showed that participants with higher levels of general concern about falling tended to have slower reaction times when cognitive task requirements increased. This relation was reduced after controlling for sensorimotor function. While this suggests that good physical abilities might be paramount in the relationship, other factors might still be at play. Shackman, Sarinopoulos [21] suggested that anxiety may interfere with visuospatial working memory, which could explain why individuals with higher levels of general concern about falling were slower during the dual-task condition. This further aligns with the attentional control theory, which proposes that anxiety interferes with attention and cognitive resources when task demands increase, possibly negatively impacting task performance [18]. In our exploratory analysis, it was also found that executive function, specifically working memory and cognitive flexibility [35, 36], had an effect on the association between general concern about falling and stepping reaction times in the dual-task condition. The relationship between cognitive function, concern about falling and stepping is consistent with previous studies that showed that poor cognitive ability was associated with both fall risk [37] and the development of concern about falling [7]. This may explain how concern about falling increases fall risk during complex activities. According to the attentional control theory it may be expected that concerns about falling will require some attentional processing during multi-tasking, which may then have detrimental effects on the efficiency of the reactive stepping performance.

### Repeated exposure to reduce task-specific concern about falling

Our results indicate that repeated exposure is beneficial to reduce task-specific concern about falling and increase confidence regarding a cognitive-motor task in participants with higher levels of concern about falling. Individuals with greater general concern about falling were less confident about whether they could do the stepping tasks. Initial lack of confidence and task-specific concern were corrected when the same stepping movement was repeated. Exposing older people to activities that they (erroneously) perceive as unsafe may increase their confidence about their ability to conduct the activity without falling, as individual’s predictions become corrected through repeated exposure. These findings are consistent with an earlier study by Rachman et al., which revealed that highly fearful individuals have an increased tendency to over-predict the value of threat [38]. As such, over-prediction of threat has been associated with avoidance of those activities which might evoke a negative outcome [39]. Such avoidance behaviour limits opportunities for individuals to experience situations which counter these irrational fears [38]. As a consequence, people might enter a negative spiral of fear-avoidance and physical deconditioning, further increasing a person’s risk of falling [11]. While our results are the first of their kind, they indicate that exposure might be successful in people with high levels of general concern about falling, but an overall good well-being, regardless of whether they had previous falls or have an increased fall risk due to reduced sensorimotor function.

### Clinical and research implications towards reducing the impact of concern about falling on fall risk

In line with previous studies, our results support the role of lowering concern about falling in improving cognitive-motor performance and potentially reduce fall risk in older people [12, 40]. We consider that a holistic approach that incorporates exercise, exposure therapy and cognitive behavioural therapy might be an effective approach to reduce concern about falling long-term. Randomised controlled trials will be required to confirm this hypothesis.

#### Exercise

The implication of the sensorimotor function affecting the relation between general concern about falling and stepping performance, is that improving physical function should be considered as a strategy. If well-designed, balance exercise programs can reduce falls by 39% as demonstrated by a comprehensive meta-analysis [41]. Exercise and physical activity are also known to improve depressed mood [42] and could improve cognitive performance in older people [43]. A Cochrane systematic review and meta-analysis showed that exercise can reduce concern about falling immediately following an intervention [44]. Exercise interventions have been proposed as a promising means for the prevention of falls and are recommended in evidence-based guidelines for fall prevention worldwide. However, the long-term effect of exercise on concern about falling is unclear [44], and might require additional strategies.

#### Graded exposure therapy

Exposure therapy may be effective in reducing short-term, task-specific concern about falling regarding perceived fall-threatening activities. To date, only one study has investigated this approach towards reducing concern about falling. Wetherell, Johnson [45] reported pilot findings showing a combined approach of exercise with exposure therapy was successful in reducing excessive levels of concern about falling. Exposure therapy has been applied successfully to reduce fear of pain and movement [46]. Similar to concern about falling, fear of pain and movement contributes to the maintenance of pain disability, which can lead to avoidance behaviour [46]. Vlaeyen, de Jong [47] implemented graded exposure, during which participants were required to perform fear-evoking activities and was successful at reducing an overall and long-term fear of pain and avoidance behaviour. Therefore, repeated exposures to pain threatening activities without suffering a negative outcome, not only decreased their fear regarding that activity but also translated to other daily activities [48]. This study showed reductions in short-term, task-specific concern about falling immediately after the performing the exposure. Future research should investigate the long-term effectiveness of graded exposure to reduce concern about falling in older people, possibly in combination with other approaches such as exercise and/or cognitive behavioural therapy.

#### Cognitive Behavioural therapy

When cognitive behavioural therapy is aimed at modifying patterns of thoughts (cognitions) and actions (behaviours) detrimental to fall risk, including avoidance behaviour [10, 49], it can also reduce concern about falling as well as the incidence of fall events [49, 50]. Such approaches might minimize the interference of concern about falling on the capacity to perform tasks requiring attentional resources, through addressing low mood as well as through addressing concern about falling directly. Furthermore, this strategy might be considered in conjunction with exposure therapy, especially in people with concomitant low mood.

#### Combined cognitive-motor training

The integration of cognitive training into established fall interventions may improve the effectiveness of current programs (e.g., exercise) to reduce fall risk in people with concern about falling. Our finding that executive function affected the relation between concern about falling and cognitive-motor performance, implies that improving working memory and cognitive flexibility [35, 36] could be effective in weakening the association between concern about falling and fall risk in more challenging cognitive-motor situations. Previous studies have observed the association between falls and executive function [15, 51], which suggests cognitive training may also contribute to the reduction of falls directly. Studies which tested the effects of cognitive training found positive results on gait, balance and fall risk [52, 53]. Therefore, cognitive training might reduce falls, in addition to reducing the negative effect of concern about falling on performance during more challenging dual-task conditions. Previous research has also shown a combination of physical and cognitive training can improve executive function [54] and reduce fall risk and concern about falling [55, 56]. A combined training approach might have stronger long-term effects compared to physical or cognitive training alone.

### Limitations

First, our sample consisted mainly of healthy older adults, and may therefore not be representative to the general older population. Second, nearly all our measures were self-report. Even though all scales and questionnaires have demonstrated validity in measuring the target outcome, response bias and shared method variance may have affected our findings. Third, the testing of exposure was a secondary objective, and can only be considered as an experimental analogue of exposure [57]. A more specific design to investigate the effects of graded exposure is required. Fourth, although we used an experimental design by manipulating the cognitive load during the stepping task, other relationships were cross-sectional in nature. We should therefore be cautious to infer causality.

## Conclusion

The study aimed to gain a better understanding of the association between concern about falling and stepping performance during complex activities. General concern about falling was associated with poorer stepping reaction times, especially when combined with a visuospatial task. Individuals with greater concern about falling tended to have longer reaction times. This association between concern about falling and stepping performance, was strongly influenced by sensorimotor function and executive function. Reduced task-specific confidence observed immediately after the task, was more pronounced in people with higher levels of general concern about falling. Multiple exposures by repetition of the stepping task improved short-term task-specific concern and confidence equally in all participants. These findings suggest that sensorimotor function, mood and executive function may be important for older people with higher levels of general concern about falling to successfully perform more complex cognitive-motor tasks.

## Data Availability

The datasets used and/or analysed during the current study are available from the corresponding author on reasonable request.

## Abbreviations

CSRT: Choice Stepping Reaction Time
FES-I: Falls Efficacy Scale International
GAS: Goldberg Anxiety Scale
GDS: Geriatric Depression Scale
PPA: Physiological Profile Assessment
TMT: Trail Making Test
